# Growth factor purification and delivery systems (PADS) for therapeutic angiogenesis

**DOI:** 10.1186/s13221-014-0026-3

**Published:** 2015-01-24

**Authors:** Eric M George, Huiling Liu, Grant G Robinson, Fakhri Mahdi, Eddie Perkins, Gene L Bidwell

**Affiliations:** Department of Physiology and Biophysics, University of Mississippi Medical Center, 2500 North State Street, Jackson, MS 39216 USA; Department of Biochemistry, University of Mississippi Medical Center, 2500 North State Street, Jackson, MS 39216 USA; Department of Neurology, University of Mississippi Medical Center, 2500 North State Street, Jackson, MS 39216 USA; Department of Neurosurgery, University of Mississippi Medical Center, 2500 North State Street, Jackson, MS 39216 USA; Department of Neurobiology and Anatomical Sciences, University of Mississippi Medical Center, 2500 North State Street, Jackson, MS 39216 USA

**Keywords:** Vascular endothelial growth factor, Elastin-like polypeptide, Drug delivery, Therapeutic angiogenesis, Purification and delivery system

## Abstract

**Background:**

Therapeutic angiogenesis with vascular endothelial growth factor (VEGF), delivered either via recombinant protein infusion or via gene therapy, has shown promise in preclinical models of various diseases including myocardial infarction, renovascular disease, preeclampsia, and neurodegenerative disorders. However, dosing, duration of expression, and tissue specificity are challenges to VEGF gene therapy, and recombinant VEGF delivery suffers from extremely rapid plasma clearance, necessitating continuous infusion and/or direct injection at the site of interest.

**Methods:**

Here we describe a novel growth factor purification and delivery system (PADS) generated by fusion of VEGF_121_ to a protein polymer based on Elastin-like Polypeptide (ELP). ELP is a thermally responsive biopolymer derived from a five amino acid repeat sequence found in human tropoelastin. _VEGF_PADS were constructed by fusion of the ELP coding sequence in-frame with the VEGF_121_ coding sequence connected by a flexible di-glycine linker. *In vitro* activity of _VEGF_PADS was determined using cell proliferation, tube formation, and migration assays with vascular endothelial cells. Pharmacokinetics and biodistribution of _VEGF_PADS *in vivo* were compared to free VEGF in mice using quantitative fluorescence techniques.

**Results:**

ELP fusion allowed for recombinant expression and simple, non-chromatographic purification of the ELP-VEGF_121_ chimera in yields as high as 90 mg/L of culture and at very high purity. ELP fusion had no effect on the VEGF activity, as the _VEGF_PADS were equally potent as free VEGF_121_ in stimulating HUVEC proliferation, tube formation, and migration. Additionally, the _VEGF_PADS had a molecular weight five-fold larger than free VEGF_121_, which lead to slower plasma clearance and an altered biodistribution after systemic delivery *in vivo*.

**Conclusion:**

PADS represent a new method of both purification and *in vivo* stabilization of recombinant growth factors. The use of this system could permit recombinant growth factors to become viable options for therapeutic angiogenesis in a number of disease models.

**Electronic supplementary material:**

The online version of this article (doi:10.1186/s13221-014-0026-3) contains supplementary material, which is available to authorized users.

## Background

Loss of VEGF signaling or increase in anti-angiogenic factors have been implicated in many diseases including preeclampsia [1], renovascular disease [2], and neurodegenerative diseases [3,4]. Therapeutic angiogenesis with supplemental VEGF administration has shown preclinical efficacy in multiple animal models [4-10]. However, due to its short plasma half-life and susceptibility to degradation, exogenous VEGF must be continuously administered, often directly at the desired site of action, to achieve therapeutic benefit. The goal of this study is to characterize a biopolymer fusion with human VEGF. Fusion with this biopolymer, a synthetic protein based on human elastin, allows for recombinant production of large amounts of the chimeric protein, very simple nonchromatographic purification, and reduced plasma clearance relative to free VEGF.

Supplemental VEGF has been supplied in several preclinical disease models by either direct administration of the recombinant protein or by gene therapy techniques. In a dog model of myocardial infarction, direct infusion of VEGF at the infarcted site improved blood flow and increased neovascular development [10]. However, daily infusion for 28 days directly at the site of the infarcted tissue was required. Similarly, continuous VEGF infusion into the myocardium for six weeks decreased the size of the ischemic zone and improved cardiac function in a swine model of myocardial infarction [8]. In renovascular disease, microvascular rarefaction associated with renal artery stenosis was associated with a marked reduction in bioavailable VEGF in the kidney [2], and intrarenal administration of VEGF improved renal function, increased microvessel density, and improved renal scarring in a swine model of renal artery stenosis [6]. In preeclampsia, increased production of the VEGF antagonist sFlt-1 has been shown to be a major driver of the maternal syndrome [11-14]. Direct administration of recombinant VEGF via continuous intraperitoneal infusion sequestered the increased circulating sFlt-1 and reduced the hypertension [5]. VEGF supplementation has also shown efficacy in preclinical models of neurodegenerative diseases. In spinocerebellar ataxia type I (SCA1), VEGF mRNA and protein levels were decreased in the Purkinje layer of SCA1 transgenic mice, and VEGF administration improved the cerebellar pathology and the motor function in these mice [4]. However, direct intracerebroventricular administration was required. Similarly, loss of hypoxia inducible VEGF in neural tissue in transgenic mice lead to degeneration of lower motor neurons, causing a syndrome similar to amyotrophic lateral sclerosis [3]. *In vitro*, VEGF protected motor neurons from apoptosis induced by several stressors, and *in vivo*, VEGF protected dorsal root ganglion neurons against paclitaxel or hyperglycemia-induced neurotoxicity [7].

These results suggest value for supplemental VEGF therapy in a myriad of disease models. However, they also highlight several drawbacks to recombinant VEGF therapy. VEGF has a very short plasma half-life. In humans, a terminal half-life of 33.7 minutes was measured after a 20 minute infusion [15]. For this reason, continuous administration, often directly at the target site, is required for efficacy. To overcome this limitation, sustained release methods have been developed. VEGF-loaded microspheres were injected into hindlimb muscles in rats, and these constructs resulted in slow release of VEGF over a period of seven days and evidence of vascular remodeling at time points as long as 70 days after a single injection [16]. In another application, VEGF-loaded microspheres were incorporated into alginate hydrogels to generate an injectable, slow-release hybrid delivery system [17]. This strategy lead to sustained release of VEGF over 28 days and marked improvement in angiogenesis and limb-sparing in a mouse model of hindlimb ischemia. In a recent report, a degradable VEGF-releasing hydrogel was created by fusing VEGF to the coagulation factor fXIIIa which was then crosslinked into fibrin hydrogels [18]. This construct exhibited a controlled degradation and VEGF release over a four week period, and yielded improved angiogenesis, perfusion, and healing in hind limb ischemia and in wound healing models.

In addition to its rapid clearance, recombinant VEGF produced in bacteria or yeast must be purified by a labor-intensive chromatographic protocol [19,20]. In a yeast expression system, yields of 40 mg of VEGF_121_ per L of culture have been reported using a nickel chromatography purification protocol [20]. However, chromatographic purification protocols are challenging to scale up to therapeutic production capacity, and the tooling required to do so contributes to increasing the manufacturing cost of goods (COGs).

Here we have developed a VEGF purification and delivery system (_VEGF_PADS) achieved by fusion of VEGF to a thermally responsive biopolymer via a flexible diglycine linker. Elastin-like polypeptide (ELP) is a genetically engineered protein consisting of a five amino acid repeating sequence [21]. This polypeptide has several unique properties that make it useful as a therapeutic delivery platform. First, it is thermally responsive, existing as a soluble protein below a characteristic transition temperature but self-associating into aggregates above that transition temperature [22]. This aggregation process is fully reversible. Second, ELPs can be expressed in bacterial recombinant expression systems, and they are easily purified due to their thermally responsive properties [23,24]. After recombinant expression and cellular lysis, ELP can be specifically separated from the soluble bacterial lysate by simply heating the solution or increasing the salt concentration in order to trigger ELP aggregation. Repeated centrifugation steps at temperatures above the ELP transition temperature leads to isolation of very pure preparations of the polypeptide. Third, because they are genetically engineered, the ELP sequence is easily modified to achieve the desired ELP size and transition temperature [22], and therapeutic proteins or peptides and targeting agents are easily fused to the ELP gene to create chimeric therapeutics [24-29]. Finally, because of its large size and its biocompatibility, ELP has many properties desired in a drug delivery vector, including a long plasma half-life [26,30], low immunogenicity [31,32], and biodegradability.

Here we show, using a simple non-chromatographic purification protocol, isolation of _VEGF_PADS as highly purified protein in yields up to 90 mg/L of bacterial culture. The purification requires only a warm water bath and a centrifuge and can be completed in 4 – 6 hours. The _VEGF_PADS retained full VEGF activity as assessed using endothelial cell proliferation, migration, and tube formation assays. Also, _VEGF_PADS exhibited a slower renal clearance and an altered biodistribution relative to unconjugated VEGF. We believe _VEGF_PADS, or growth factor PADS in general, represent a new, simple way to purify recombinant growth factors. They have the potential, either as stand alone agents or in combination with the novel controlled release methods recently described, to function as stabilized agents for therapeutic angiogenesis.

## Methods

### Generation of constructs

The coding sequence for VEGF_121_ was synthesized with codons optimized for expression in *E. coli* (Life Technologies), and inserted into a plasmid vector between NdeI and BamHI restriction sites, with an SfiI site at the N-terminus of the VEGF_121_ coding sequence. The entire coding sequence was cloned into pET 25b + at the NdeI and BamHI sites, and the ELP coding sequence was excised from pUC19-ELP and cloned into the SfiI site, generating an in-frame fusion of ELP and VEGF_121_. The ELP sequence contained 160 VPGxG repeats in which the x residue was V, G, or A in a 1:7:8 ratio. All constructs were confirmed by DNA sequencing.

### Purification of _VEGF_PADS

pET25b + vectors containing the _VEGF_PADS coding sequence were transformed into *E. coli* BLR(DE3), and 500 mL cultures were grown for 16 – 20 hours in 2L flasks. The pET system produces low-level recombinant protein expression even without induction [33]. Cells were harvested by centrifugation, lysed by sonication, and nucleic acids were precipitated with polyethyleneimine and removed by centrifugation. NaCl was added to the soluble lysate to a concentration of 200 mg/mL, and the solution was heated at 42°C until the _VEGF_PADS precipitated. The precipitated _VEGF_PADS were collected by centrifugation, re-dissolved in cold PBS, centrifuged at 4°C to remove any un-dissolved precipitate, and this heat cycling process was repeated 3 – 5 times until purified protein was obtained. Purity was assessed by SDS-PAGE.

### Cell culture

Human umbilical vein endothelial cells (HUVECs) were obtained from ATCC and maintained in M200 medium plus low serum growth supplement (Life Technologies) in a humidified 37°C incubator at 5% CO_2_. All experiments were performed on cells with <10 passages in culture. Cells were removed from flasks by trypsinization and counted using a Scepter® hand held cytometer (Millipore).

### HUVEC proliferation assay

HUVECs were plated in 96 well plates (10,000 cells/well). Cells were serum and growth factor starved for 24 h in M200 medium without supplements then exposed to the indicated concentration of VEGF_121_ (ProSpec) or _VEGF_PADS for 72 h. Cell number was determined using the MTS aqueous cell proliferation assay (Promega). Experiments were performed in quadruplicate, and the data represent the mean ± s.e. of 3 independent experiments.

### HUVEC tube formation assay

Sterile, non-tissue culture treated 24 well plates were coated with growth factor reduced Matrigel (BD Biosciences). 50,000 growth factor starved HUVECs were added per well in M200 growth medium + 0.1 mg/mL heparin without serum growth supplements, and PBS vehicle control, ELP control, VEGF_121_, or _VEGF_PADS were applied at a final concentration of 20 nM. Cells were incubated for 6 h at 37°C, then cells were imaged with an inverted brightfield microscope and 10× magnification objective. Five non-overlapping fields were imaged per well, and the number of tubes per field were counted and averaged for each well. Only tubes connecting two cell nodes were counted. Data represent the mean ± s.e. of three independent experiments.

### HUVEC migration assay

HUVECs (30,000 cells/well) were placed in the upper well of Boyden chambers with 8 μm membrane pores coated with Matrigel (Corning BioCoat™) in M200 medium + 1% fetal bovine serum + 0.1 mg/mL heparin. The lower chamber contained identical medium plus PBS vehicle control, ELP control, VEGF_121_, or _VEGF_PADS at a final concentration of 10 nM or 50 nM. Cells were incubated for 16 h at 37°C. The cells on the upper surface of membranes were scratched off using cotton Q-tips. Membranes were removed, stained with 0.1% crystal violet in 10% ethanol, and the number of cells on the lower membrane surface were counted in four independent fields per membrane. Experiments were performed in duplicate, and data represent the mean ± s.e. of three independent experiments.

### Polypeptide labeling

VEGF_121_ (ProSpec) or _VEGF_PADS were dissolved at 100 μM in 0.1 M NaHCO_3_ buffer, pH 8.3, and Alexa Fluor 633® succininimidyl ester (Life Technologies) was added to a final concentration of 300 μM. The reaction was allowed to proceed for 1 h at room temperature, then unreacted dye was removed by multiple washes with an Amicon 3,000 molecular weight cutoff spin filter (Millipore). Labeling efficiency was determined spectrophotometrically using a method modified from [24]. Removal of unreacted label was confirmed by TCA precipitation of the labeled protein and assessing the free fluorophor levels in the supernatant spectrophotometrically.

### Pharmacokinetics and biodistribution

All animal use was approved by the Institutional Animal Care and Use Committee at the University Of Mississippi Medical Center and was carried out in accordance with the National Institutes for Health Guide for the Care and Use of Laboratory Animals. All procedures were carried out under full surgical isoflurane anesthesia. C57Bl/6 mice were catheterized in the femoral artery, and 123 nmol/kg AlexaFluor 633 – labeled VEGF_121_ or _VEGF_PADS were injected in the opposite femoral vein. Blood was sampled intermittently for a period of four hours. Four hours after injection, the animals were euthanized and the tissues removed for *ex vivo* fluorescence analysis.

Plasma fluorescence was determined by direct measurement of the fluorescence intensity of 2 μL plasma samples with 610 nm excitation and 660 nm emission using a fluorescence plate reader and a Nanoquant® plate (Tecan). Standard curves of the injected proteins were produced using known quantities of the injectate, and standards were scanned using the same scan settings as were used for plasma samples. Plasma fluorescence intensity was fit to the standard curves to determine the molar plasma concentration at each time point, and data were averaged for all animals (n = 4 mice per group) and represented as mean ± s.d. Averaged plasma clearance data were fit to a two compartment pharmacokinetic model as described previously [25].

Whole organ *ex vivo* fluorescence imaging was performed using an IVIS Spectrum (Caliper Life Sciences, Perkin Elmer) with 605 nm excitation, 660 nm emission, and auto exposure. Mean fluorescence radiant efficiency was determined for each organ using Living Image Software (Caliper). 100 μL of each protein standard were placed in wells of a black 96 well plate and imaged with the same settings as were used for tissue imaging. Background autofluorescence from tissues of uninjected animals was subtracted from each organ’s fluorescence, and mean fluorescence radiant efficiency of all organs were fit to the standard curve values to determine tissue concentrations. Data were averaged for all animals (n = 4 mice per group) and represented as mean ± s.e.

#### Plasma stability and Dye release

Stability of _VEGF_PADS to proteolysis and stability of the chemically linked fluorescent dye were determined by *in vitro* incubation in mouse plasma. _VEGF_PADS were labeled with 5-(and-6)-carboxytetramethylrhodamine succinimidyl ester (Life Technologies) as described above. Fluorescently labeled _VEGF_PADS were diluted 1:2 from a 200 μM stock in 100% mouse plasma and incubated up to 24 h at 37°C. At the end of the incubation period, samples were added to SDS-PAGE loading dye, heated at 95°C for 5 minutes, and electrophoresed on a 4 – 20% gradient gel under non-reducing conditions. Control samples of _VEGF_PADS in PBS and _VEGF_PADS in plasma with no 37°C incubation (immediately mixed with PAGE loading dye) were also run. The gel was imaged using an IVIS Spectrum in fluorescence mode with 535 nm excitation, 580 nm emission and a 1 minute exposure time. To calculate protein degradation, total band intensity (total fluorescent radiant efficiency) for the entire lane and intensity of all bands < 50 kDa were measured. The percentage of the total band intensity <50 kDa was determined and expressed relative to the 0h incubation.

To detect dye release from the labeled proteins, an aliquot of the same protein/plasma mixture from each time point were measured directly to detect the total fluorescence using a Nanoquant® plate and a fluorescence plate reader with 543 nm excitation and 575 nm emission and a gain value of 90. After measuring the total fluorescence, the protein component of each sample was precipitated by 1:1 mixture with 10% trichloroacetic acid (TCA) and centrifugation for five minutes at 13,000 × g. The fluorescence of the supernatant, containing any non-protein bound fluorophor, was measured using the same settings. After correction for dilution, the percentage of non-protein bound fluorescence was calculated and expressed as percent free dye.

### Statistical analysis

Proliferation data were assessed using a two way ANOVA for polypeptide agent and concentration factors, and a Bonferroni multiple comparison was performed. Tube formation and migration data were assessed using a one-way ANOVA with a *post hoc* Bonferroni multiple comparison to compare treatment groups. Differences in urine levels were compared using a Student’s t-test. Organ biodistribution was assessed with a two way ANOVA for factors of polypeptide treatment and organ type, and a Bonferroni multiple comparison was used to assess significant differences. In all analyses, a p value of < 0.05 was considered statistically significant.

## Results and discussion

### Purification and activity of _VEGF_PADS

**Figure 1:**
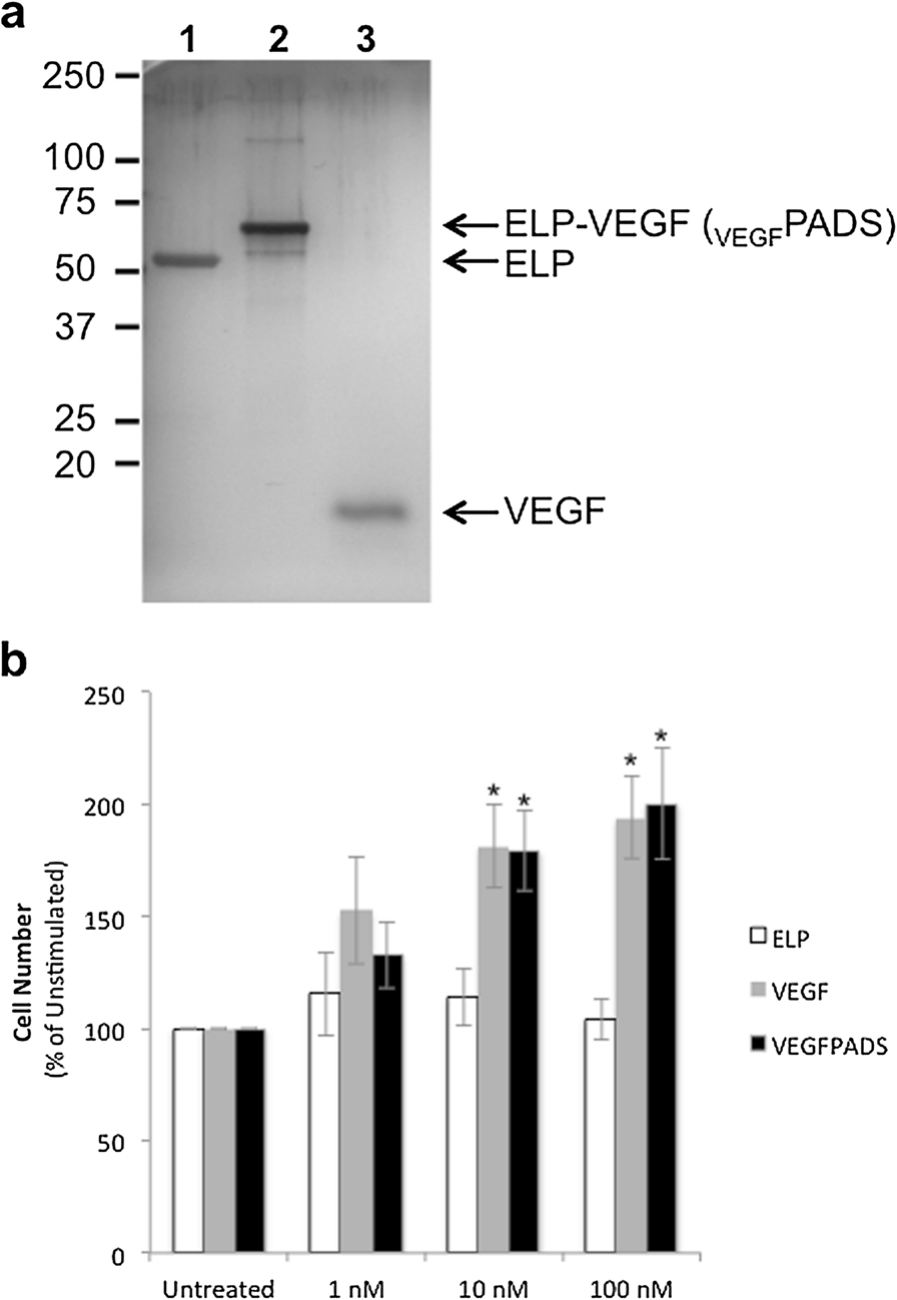
**Purification and activity of**_**VEGF**_ **PADS. a**. SDS-PAGE gel with silver staining demonstrating the purity of _VEGF_PADS and ELP control polypeptides. Lane 1, ELP; Lane 2, _VEGF_PADS; Lane 3, VEGF_121_. **b**. HUVEC cell proliferation was determined after 72h exposure to ELP, VEGF, or _VEGF_PADS at the indicated concentrations using the MTS cell proliferation assay. *Statistically significant increase versus untreated cells (p = 0.0003, two-way ANOVA with post-hoc Bonferroni multiple comparison).

The coding sequence for VEGF_121_ was cloned into a pET expression vector in frame with the ELP coding sequence to enable recombinant production. The chimeric ELP-fused VEGF (_VEGF_PADS) was purified by taking advantage of the thermally responsive property of the ELP moiety. After bacterial lysis, the _VEGF_PADS were separated from other soluble proteins by increasing the salinity of the solution and raising the temperature, which induces a reversible aggregation of the ELP domain. Centrifugation under these conditions selectively precipitated the _VEGF_PADS, and they were re-solubilized by mixing in cold phosphate buffered saline. As shown in Figure 1a, three thermal precipitation cycles were sufficient to produce highly purified _VEGF_PADS, and they migrated on a polyacrylamide gel at approximately the expected molecular weight. This method routinely yielded at least 90 mg of _VEGF_PADS per liter of bacterial culture, and the entire purification protocol from lysis to pure protein could be accomplished in less than one day.

We next assessed whether the _VEGF_PADS maintained their activity. It is possible that fusion of VEGF to the larger ELP domain could affect its receptor binding and reduce or eliminate its potency. To assess whether _VEGF_PADS could stimulate proliferation of endothelial cells, HUVECs in culture medium in the absence of growth factors were exposed to a concentration range of either free VEGF or _VEGF_PADS. _VEGF_PADS were very potent stimulators of HUVEC proliferation, achieving significant enhancement of proliferation rate at a concentration of as low as 10 nM (Figure 1b). Importantly, the potency of _VEGF_PADS was equivalent to that of free recombinant VEGF_121_ in this proliferation assay.

**Figure 2:**
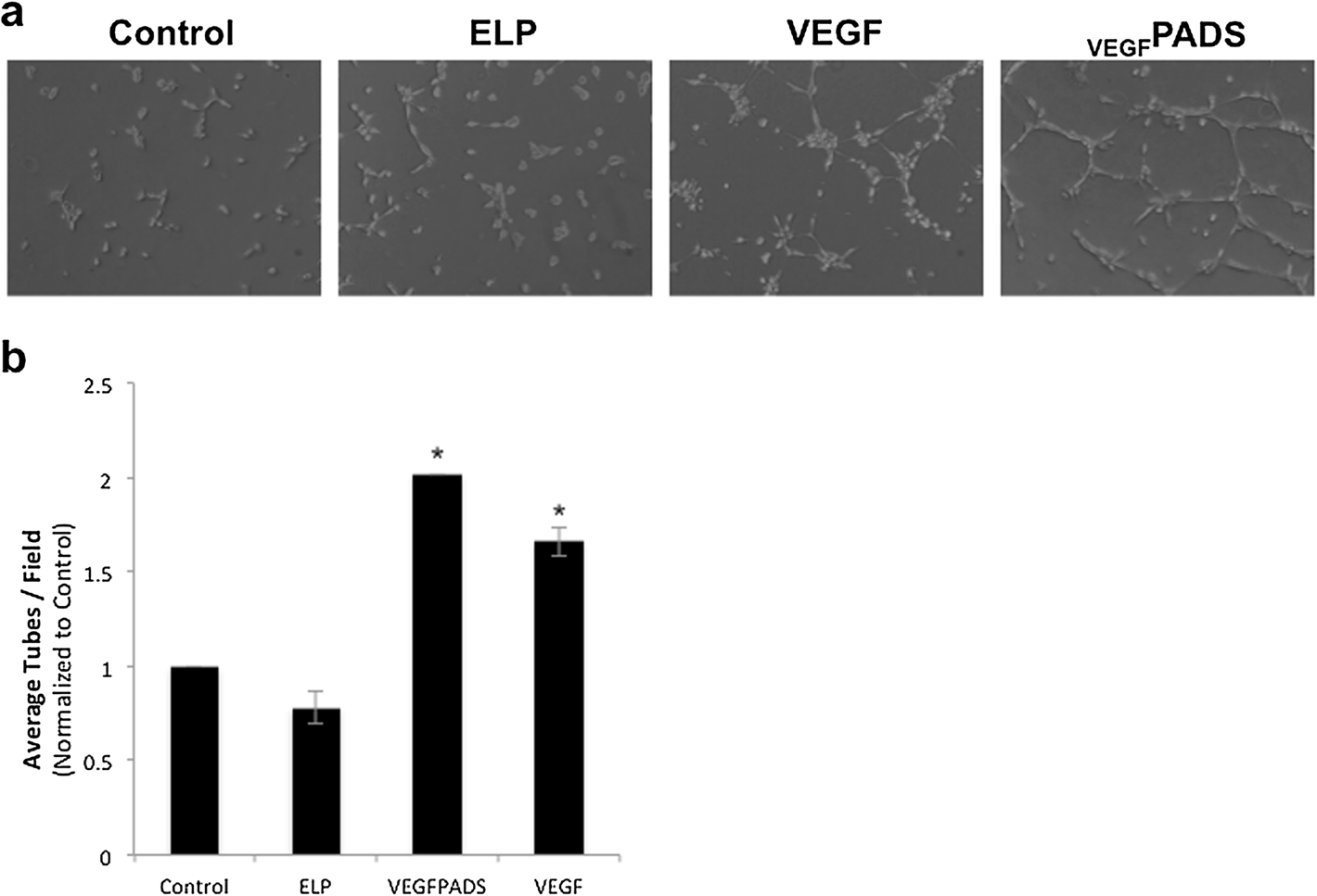
_**VEGF**_ **PADS stimulate tube formation in HUVECs. a**. HUVEC tube formation was assessed 6 h after seeding on growth factor reduced Matrigel and supplementing the media with 20 nM ELP, VEGF, or _VEGF_PADS. **b**. Average tubes per field were counted for six fields per sample. Data represent the mean ± se of four independent experiments. *p = 0.000003, one-way ANOVA with post-hoc Bonferroni multiple comparison.

**Figure 3:**
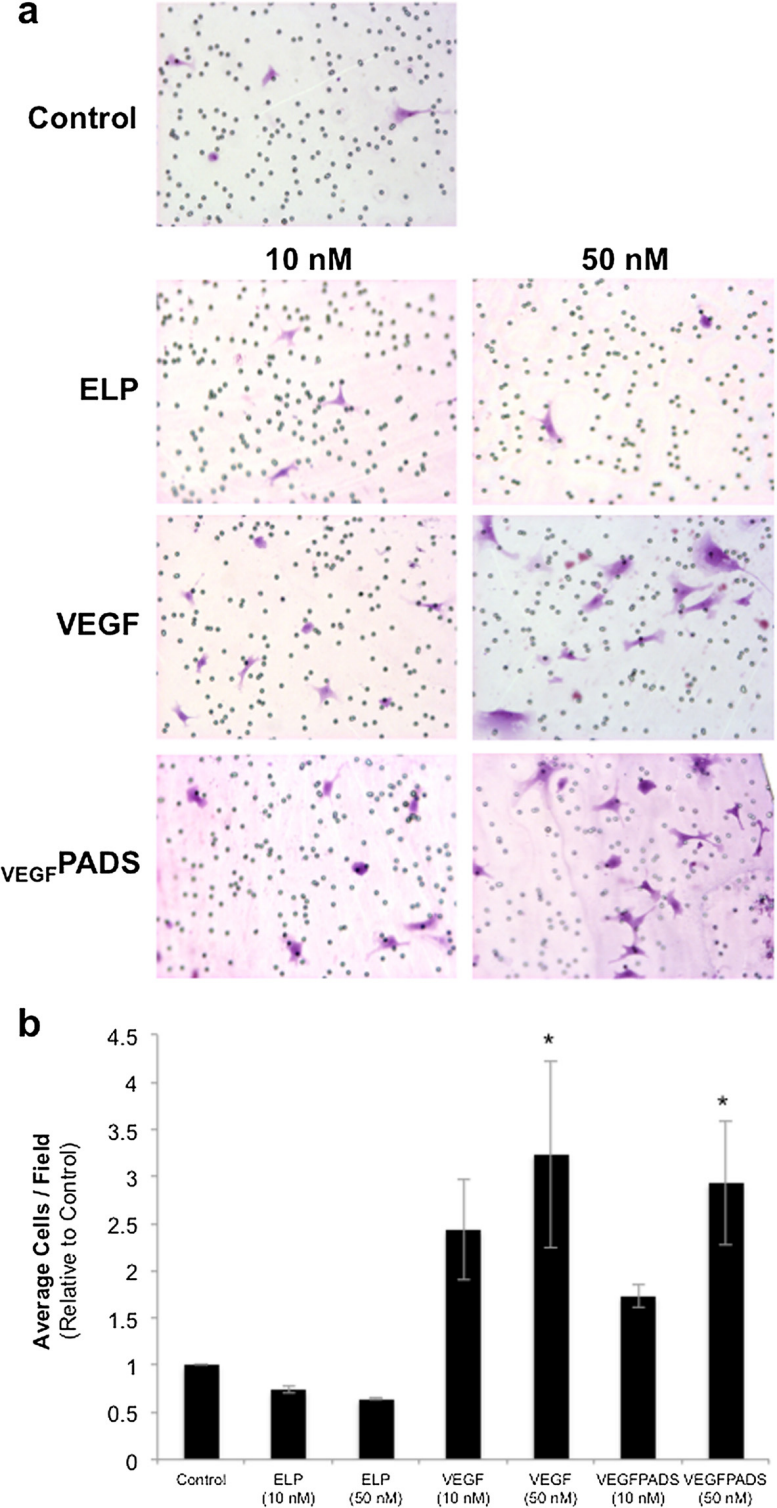
_**VEGF**_ **PADS stimulate HUVEC migration. a**. HUVEC migration was assessed 16 h after seeding in the top chamber of Matrigel-coated Boyden chambers in minimal media and supplementing the bottom chamber minimal media with ELP, VEGF, or _VEGF_PADS at the indicated concentrations. **b**. Average cells per field were counted for four to seven fields per sample. Data represent the mean ± se of three independent experiments. *p = 0.002, one-way ANOVA with post-hoc Bonferroni multiple comparison.

We also tested the ability of _VEGF_PADS to stimulate endothelial cell tube formation and migration. Tube formation was assessed using a growth factor reduced Matrigel assay. When plated on growth factor reduced Matrigel, HUVECs did not efficiently form tubes (Figure 2a, left panel). However, when the medium was supplemented with either free VEGF_121_ or _VEGF_PADS, tube formation was stimulated (Figure 2a, right panels). The ELP protein alone without VEGF fusion had no effect on tube formation. Tubes were counted for each treatment group, and this analysis revealed that _VEGF_PADS were equally as potent as free VEGF_121_ at stimulating HUVEC tube formation (Figure 2b). In addition to tube formation, _VEGF_PADS also stimulated HUVEC migration. As shown in Figure 3a, both free VEGF_121_ and _VEGF_PADS stimulated HUVEC migration through Matrigel in a Boyden chamber invasion assay. Both proteins stimulated migration at concentrations as low as 10 nM and produced equipotent and statistically significant migration at 100 nM (Figure 3b).

### Pharmacokinetics and biodistribution of _VEGF_PADS versus free VEGF

**Figure 4:**
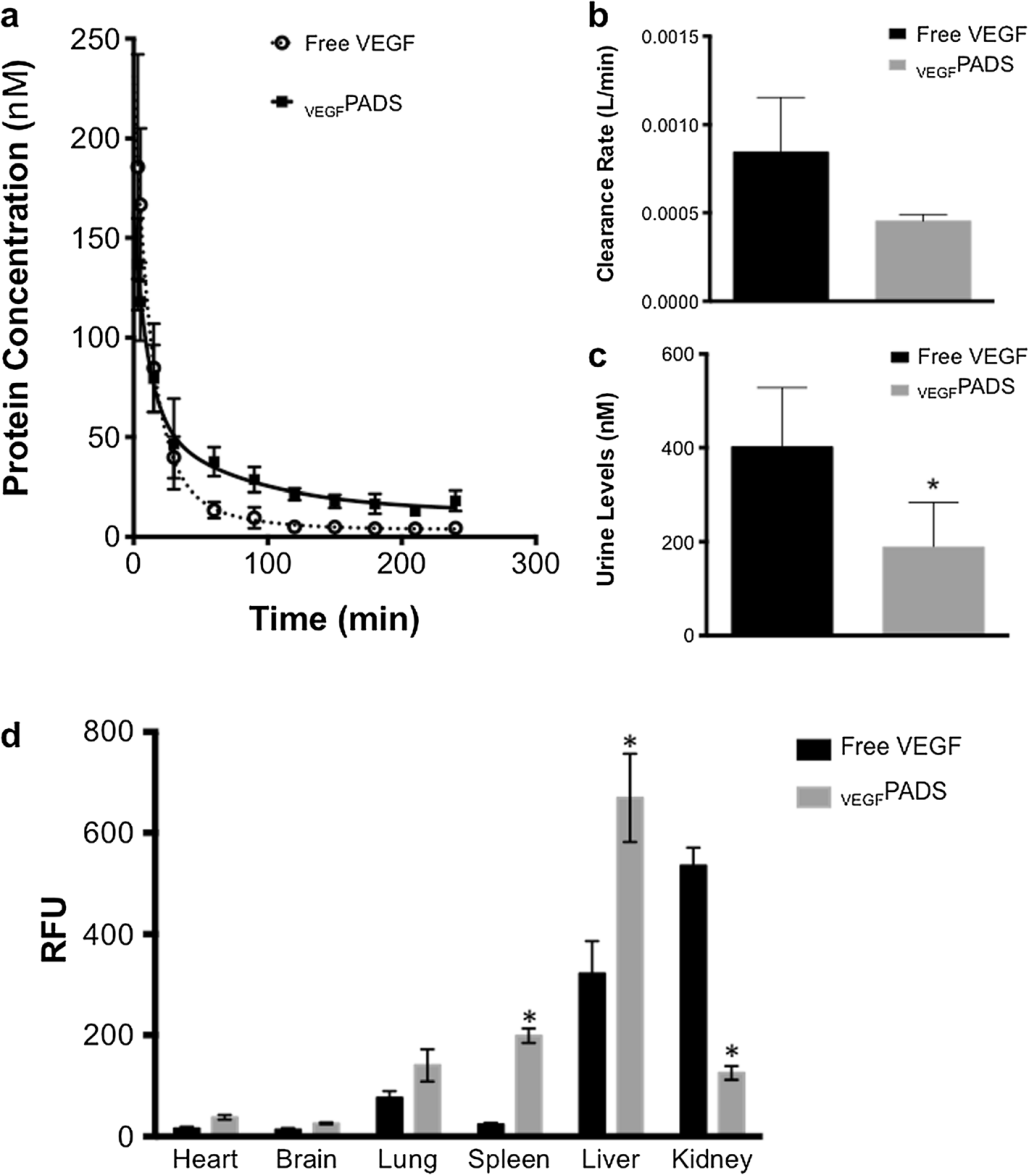
_**VEGF**_ **PADS pharmacokinetics and biodistribution. a**. Fluorescently labeled free VEGF or _VEGF_PADS were administered by IV injection to C57/Bl6 mice. Plasma levels were determined by direct fluorescence quantitation and fit to a two-compartment pharmacokinetic model. **b**. _VEGF_PADS had a slower plasma clearance rate than free VEGF, as was evidenced by lower levels in the urine at the end of the experiment **c**. Data represent the mean ± sd of four mice per group. *p = 0.03, Student’s t-test. **d**. ELP fusion significantly altered the biodistribution of VEGF, increasing its levels in the spleen and liver and reducing its levels in the kidney. *p <0.05, two-way ANOVA with post-hoc Bonferroni multiple comparison.

In addition to examining the _VEGF_PADS activity *in vitro*, we also determined the pharmacokinetics (PK) and biodistribution of _VEGF_PADS in comparison to free VEGF_121_. Both free VEGF_121_ and _VEGF_PADS were fluorescently labeled, and their PK and biodistribution were determined in mice after bolus intravenous administration. Free VEGF_121_ had a very rapid plasma clearance (Figure 4a), and fitting to a 2-compartment PK model revealed a terminal plasma half-life of approximately 30 minutes (Table 1). This is consistent with other reports of approximately a 30 minute half-life for recombinant VEGF in humans [15]. _VEGF_PADS cleared more slowly than free VEGF_121_ (Figure 4a). Their plasma clearance rate after IV infusion was about half the rate of free VEGF_121_ (Figure 4b and Table 1), and as a result, there was less fluorescence detectable in the urine at the end of the experiment (Figure 4c). Four hours after the infusion, the biodistribution was determined by *ex vivo* whole organ fluorescence imaging. VEGF_121_ accumulated most highly in the kidneys and the liver and had very low levels in other organs. In contrast, _VEGF_PADS accumulated more highly in the spleen and liver than did free VEGF_121_, and the kidney deposition of _VEGF_PADS was significantly lower than for free VEGF_121_ (Figure 4d).

**Table 1:** **Pharmacokinetic parameters of VEGF**_**121**_ **and**_**VEGF**_ **PADS**

			**Free VEGF**	_**VEGF**_ **PADS**
Central compartment volume of distribution	*V*_*c*_	(L)	0.013 ± 0.005	0.013 ± 0.003
Plasma clearance	*Cl*	(L min^-1^)	0.0008 ± 0.0003	0.0004 ± 0.0004
Area under curve	*AUC*	(nmol min L^-1^)	3,863.8 ± 1,444.6	5,059.0 ± 528.5
Distribution half life	*t*_*1/2 dist*_	(min)	8.6	7.2
Terminal half life	*t*_*1/2 term*_	(min)	30.2	52.4

We also confirmed that the pharmacokinetic and biodistribution results were not influenced by release of the dye from the carrier or by significant _VEGF_PADS degradation. Using SDS-PAGE analysis of _VEGF_PADS incubated in plasma at 37°C *in vitro* for up to 24 h (Additional file 1: Figure S1a), we determined that the protein undergoes a slow degradation. By measuring the percentage of fluorescence present in bands less than 50 kDa, _VEGF_PADS degraded at a rate of about 10% per day (Additional file 1: Figure S1b). This value is consistent with our observations of related ELP-based proteins in a rat model *in vivo* [34] and with the rate observed for the parent ELP carrier in a mouse model using a radiolabeling technique [35]. Also, the rate of degradation is much slower than the plasma clearance rate, indicating that protein degradation is not a major factor on the time scale of the *in vivo* pharmacokinetic measurements. Protein biodegradation is likely the mechanism by which _VEGF_PADS are eventually cleared from deposits within tissues, but this process will occur over a matter of days. Finally, by measuring the fluorescence of the protein/plasma mixture before and after TCA precipitation, we observed that less than 2% of the fluorophor was released from the protein over the course of a 24 h incubation in plasma (Additional file 1: Figure S1b).

In summary, by fusing VEGF to an engineered polypeptide carrier, we created a chimeric protein that is very easily purified from a recombinant expression system and maintains full VEGF activity as assessed in human endothelial cells. The _VEGF_PADS system allows for production of gram quantities of the recombinant growth factor at very high purity with a very simple purification scheme. This could represent a mechanism to facilitate the production of growth factors in a fast and inexpensive manner to make them more accessible for research purposes or to produce them at the scale needed for therapeutics. The system can easily be modified for the production of other growth factors, and we are in the process of generating other VEGF isoforms as ELP fusions as well as other _GF_PADS using several growth factors of interest for various diseases. In addition to maintaining their signaling ability, _VEGF_PADS also showed extended plasma life and altered biodistribution compared to the free growth factor. Given their ease of production, their potency, and their increased *in vivo* stability, _VEGF_PADS could prove to be useful therapeutics, either as standalone agents or in combination with the controlled release strategies described above.

We are currently evaluating the therapeutic efficacy of _VEGF_PADS in several disease models in which decreased VEGF levels have been implicated. For example, in preeclampsia, a major contributor to the maternal hypertension and other symptoms is the VEGF antagonist sFlt-1 [11]. We have recently described the ability of the ELP carrier to prevent placental transfer and fetal exposure of attached cargo [34], and we are evaluating the ability of _VEGF_PADS to bind the excess sFlt-1 and restore the available VEGF levels while preventing fetal exposure to VEGF in a rodent preeclampsia model. Also, VEGF has been demonstrated to increase microvascular density and partially restore renal function in a swine model of renovascular hypertension [6]. We are currently evaluating the ability of _VEGF_PADS to increase the VEGF bioavailability after systemic or direct intrarenal administration and restore or preserve renal function. Finally, VEGF has been shown to be reduced in the Purkinje layer of the cerebellum in the neurodegenerative disease SCA1 [4]. We are currently evaluating the brain deposition, clearance kinetics, and therapeutic efficacy of _VEGF_PADS versus unconjugated VEGF in a genetically engineered mouse model of SCA1.

## Conclusion

The work presented here establishes _VEGF_PADS as easily produced, highly active modifications of recombinant growth factors. The ELP system is easily amenable to modification with any desired proteinacious therapeutic, and thus _VEGF_PADS are but one example of a therapeutic that can be generated using the ELP-based PADS. Given the ease of purification and the *in vivo* stabilization conferred by ELP fusion, we believe _VEGF_PADS have great potential for therapeutic angiogenesis in a variety of disorders.

## Additional file

Additional file 1: Figure S1. Protein Stability and Dye Release in Plasma. **a**. The rate of degradation of VEGFPADS was determined after incubation for various times in mouse plasma at 37°C by SDS-PAGE analysis. **b**. The percentage of the total band intensity less than 50 kDa was determined for each time point (left axis). Also, dye release was detected by measuring the total plasma fluorescence before and after TCA precipitation of the protein component (right axis).

## Abbreviations

ELP: Elastin-like polypeptides
HUVEC: Human umbilical vein vascular endothelial cells
PADS: Purification and delivery system
SCA1: Spinocerebellar ataxia type I
SDS-PAGE: Sodium dodecyl sulfate polyacrylamide gel electrophoresis
TCA: Trichloroacetic acid
VEGF: Vascular endothelial growth factor
_VEGF_PADS: VEGF Purification and delivery system
