# Supplementary material for: Growth factor purification and delivery systems (PADS) for therapeutic angiogenesis
Source: Vasc Cell. 2015 Jan 24;7(1):1. doi: 10.1186/s13221-014-0026-3 (PMC4316602; doi:10.1186/s13221-014-0026-3)
Supplement: Additional file 1: Figure S1. — Protein Stability and Dye Release in Plasma. a. The rate of degradation of VEGFPADS was determined after incubation for various times in mouse plasma at 37°C by SDS-PAGE analysis. b. The percentage of the total band intensity less than 50 kDa was determined for each time point (left axis). Also, dye release was detected by measuring the total plasma fluorescence before and after TCA precipitation of the protein component (right axis). [file 13221_2014_26_MOESM1_ESM.pdf]

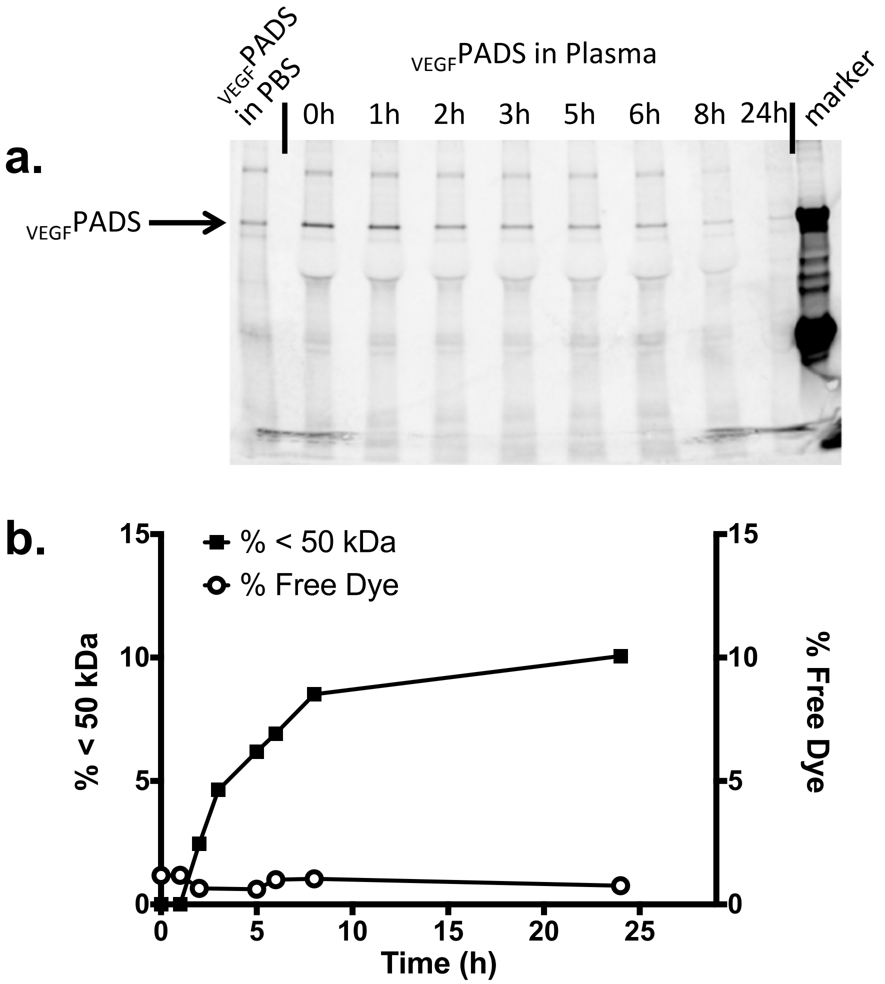

Supplementary Figure 1. Protein Stability and Dye Release in Plasma. **a.** The rate of degradation of $_{VEGF}$PADS was determined after incubation for various times in mouse plasma at 37 °C by SDS-PAGE analysis. **b.** The percentage of the total band intensity less than 50 kDa was determined for each time point (left axis). Also, dye release was detected by measuring the total plasma fluorescence before and after TCA precipitation of the protein component (right axis).
